# Prevention and control of HPV infection and HPV-related cancers in Colombia- a meeting report

**DOI:** 10.1186/s12919-020-00192-2

**Published:** 2020-06-22

**Authors:** Alex Vorsters, Francesc Xavier Bosch, Paolo Bonanni, Eduardo L. Franco, Marc Baay, Clarissa Simas, Dur-e-Nayab Waheed, Carlos Castro, Raul Murillo, Lina Trujillo, Carolina Wiesner, Nubia Muñoz

**Affiliations:** 1 Centre for Evaluation of Vaccination, Vaccine and Infectious Diseases Institute, University of Antwerp, Universiteitsplein 1, 2610 Wilrijk, Antwerp, Belgium; 2 Cancer Epidemiology Research Program, IDIBELL, Catalan Institute of Oncology, L’Hospitalet De Llobregat, Barcelona, Spain; 3 Open University of Catalonia, Barcelona, Spain; 4 University of Florence, Health Sciences, Florence, Italy; 5 Department of Oncology, McGill University, Montreal, Canada; 6 P95, Epidemiology and Pharmacovigilance Consulting and Services, Leuven, Belgium; 7 Infectious Disease and Epidemiology (IDE), London School of Hygiene and Tropical Medicine, London, UK; 8 Colombian League against Cancer Bogota, Bogota, Colombia; 9 Centro Javeriano de Oncología, Hospital Universitario San Ignacio, Bogota, Colombia; 10 Instituto Nacional de Cancerología, Bogota, Colombia

**Keywords:** Human papilloma virus, HPV, Vaccination, Cervical cancer prevention, Screening

## Abstract

The Human Papillomavirus (HPV) Prevention and Control Board is an independent multidisciplinary board of international experts that disseminates relevant information on HPV to a broad array of stakeholders and provides guidance on strategic, technical and policy issues in the implementation of HPV control programs. In response to drastic drop of vaccine coverage following the adverse event crisis in Carmen del Bolivar, Colombia, the HPV Prevention and Control Board in collaboration with the Colombian National Cancer Institute and Colombian League Against Cancer convened a meeting in Bogota, Columbia (November 2018). The goal of the meeting was to bring together national and international group of experts to report the disease burden, epidemiology and surveillance of HPV and HPV-related cancers, to discuss the successes and especially the challenges of HPV vaccination and screening in Colombia, as well as the lessons learnt from neighbouring countries. The meeting provided a platform to confer various stakeholder’s perspectives, including the role of the Colombian healthcare system and to catalyse various parts of the public health community in Colombia into effective action. The conclusion of the meeting included following suggestions to strengthen HPV prevention and control: 1) Re-introducing school-based vaccine programs, 2) Integrating primary and secondary prevention programs, 3) Developing an innovative crisis communication plan targeting healthcare workers, teachers and general population, 4) Building trust through efficient and timely communication, 5) Building strong relationship with media to ensure a stable vaccination campaign support, and 6) Promoting empathy among healthcare professionals towards patients to build trust and communicate effectively.

## Introduction

The HPV Prevention and Control Board (www.hpvboard.org) [1] is an international independent multidisciplinary board created in 2015 by the prominent experts in the field. The HPV Board aims at being the important catalyst to multiply and disseminate relevant information on HPV prevention and control to a broad array of stakeholders. The board is comprised of a prominent group of experts serving as advisors to the board who provide evidence based reflection and guidance on strategic, technical and policy issues that occur as we forward in the implementation of HPV control programs. It contributes to control of HPV infection, prevention and screening strategies of HPV related cancers by organizing two meetings per year; 1) a technical meeting covering topics such as vaccine characteristics, vaccine safety, screening technologies and landscape, treatment strategies, dealing with anti-vaccine messages, and 2) a country meeting covering SWOT analysis of a country or region. The HPV Board convened its sixth meeting in Bogota, Colombia on November 15–16, 2018 to address drastic drop of vaccine coverage due to reported adverse events following HPV vaccination in a school-based programme in Carmen del Bolivar, Colombia. The crisis resulted in vaccine uptake among eligible girls declined to 14% for the first dose and 5% for the complete course, down from 98 and 88%, respectively, in 2012 [2]. The objectives of the meeting were: understanding epidemiology, burden of disease and surveillance related to HPV and HPV related cancers in Colombia; discussing successes, topical issues and challenges related to HPV vaccination and screening particular to Colombia; and with a focus on the current crisis situation reviewing country examples from Latin America to learn from their successes and challenges; gaining insight into various stakeholder perspectives; and propose recommendations for the way forward; and advise other countries how to deal with these crises when they occur. Germane to the discussion were presentations on the Colombian health care system, to shed light on possible roles that it may have in averting vaccine confidence issues. An important challenge for Colombia is to recover the HPV vaccination coverage to levels before the Carmen de Bolivar crisis in 2014. International and local experts discussed all aspects that could enhance HPV vaccine uptake. However, this also provided an opportunity to discuss optimization of cervical cancer screening efforts in Colombia. This report summarizes the discussions and lessons learned from the participants.

## The healthcare system in Colombia

Colombia is divided into 32 departments and one capital district, each with its own governor.

It has a population of approximately 45.5 million inhabitants, unevenly dispersed over the country. This is directly related to the geographical diversity of the country, including a mountain range, coastal regions, plains and the Amazon rainforest. Together, the lowlands comprise over 50% of the territory, but contain approximately 5% of the population. Moreover, 76% of the population live in urban areas. The age composition of Colombia is approximately 23% aged < 18, 68% aged 18–65 and 9% > 65 years. The life expectancy is 76 years and is slightly higher in females than in males. In 2017, the National Administrative Department of Statistics reported that 27% of the population was living below the poverty line, of which 7% of the population lived in extreme poverty.

Health insurance comes in two forms: a subsidized system for people with low income, and a contributing system for those who have a job and make monthly payments to the system. An estimated 97% of the population is covered by health insurance that provides broad benefits for all ages and all incomes. Nationally, the cost of healthcare is between 6 and 8% of the gross domestic product. It is a very complex healthcare system that has three main actors: the territories or political entities (municipalities and departments), the Entidades Promotoras de Salud (EPS) are the insurance companies that can be public or private and the Instituciones Prestadores de Salud (IPS) are the healthcare providers (hospitals and clinics) which mainly are privately owned. Each person can choose his/her EPS. The Ministry of Health coordinates the actions of these sectors.

The major problems in the Colombian healthcare system are: over-consumption, too many procedures; fragmentation due to an abundance of private insurers, lack of coordination between the basic health care providers and the complementary ones; lack of third-level care; high cost of administration; lack of qualified personnel; inequality in care; and systematic focus on treatment of disease rather than disease prevention [3–5].

A new health system model has been proposed that rethinks the health system from one based on performance and payment of services to a model around people; it reorients the health system towards holistic health care, which does not focus on disease but promotes health and well-being of people at the level of primary and community care, as well as at levels of greater complexity. This model proposes the creation of health networks and the production of knowledge in health and well-being, whose positive results have been demonstrated in Colombia by Compensar EPS [6]. This insurer contracted hospitals, clinics, primary care teams and other health-related providers to deliver the services needed by the population it had committed to serve. Together, they developed a model to integrate their activities and align their incentives, ensuring that every person had a family physician, which is supported by community-based providers, with access to a good hospital environment and home care, while being enabled by a leading-edge information system.

## Epidemiology of HPV and cervical cancer in Colombia

### The role of Colombia in HPV epidemiology research

Colombia has a rich history of research on HPV epidemiology and public health, mostly performed by the International Agency for Research on Cancer (IARC, Lyon, France), in collaboration with the Vale University, the Colombian Cancer Institute and the Catalan Institute of Oncology (ICO). This includes the first population-based case-control study that established the causal association between HPV and cervical cancer, performed in Cali and in Spain including 400 cervical cancer cases and 450 controls [7], followed by a larger study conducted in 11 countries that included 2500 cervical cancer cases and 2500 controls [8], This study proposed an epidemiological classification of HPV types, in high-risk, intermediate-risk and low-risk types and estimated that HPV16 and 18 were responsible for 70% of cervical cancers and HPV 31, 33, 45 52 and 58 for an additional 20%. Colombia also participated in one of the first multi-site studies investigating the male role in HPV transmission and the risk of cervical cancer. This study showed that penile HPV prevalence was high in both husbands of women with cervical cancer and women from control group, in all countries except Spain where husbands of women with cervical cancer had a higher penile HPV prevalence than husbands of women from control group. Furthermore, the protective effect of circumcision was shown in circumcised men, HPV prevalence was much lower in penile swabs [9]. Colombia participated, as one of 22 countries, in the International Prevalence Survey that established HPV as necessary cause of cervical cancer [10], and provided samples from 1800 women for the IARC International Survey on HPV types in women without cervical cancer [11]. This study reported an HPV DNA prevalence of 15% in Colombian women aged 15 to 64 years. Colombian samples from cervical cancer, other genital cancers and Head & Neck (H&N) cancer cases were also included in the ICO International Survey on HPV-associated cancers [12].

Finally, to investigate the natural history of HPV, a cohort study was performed in Bogota [13], that followed approximately 2000 women, aged 15 to 80 years, every 6 months for 10 years. This study showed the prevalence and incidence of type-specific infection with HPV in women with normal cytology and women with cervical neoplasia. The incidence of high-risk types was higher than that of low-risk types (5.0 vs. 2.0 cases/100 woman-years), and infections with high-risk types lasted longer, on average, than infections with low-risk types (14.8 vs. 11.1 months) [13]. All above studies conducted in Colombia, were a key element in the decision of the Colombian government to introduce the HPV vaccine in the national immunization program.

### Burden of HPV associated cancers in Colombia

In Colombia, cervical cancer is by far the most prominent HPV-associated cancer (Table 1). However, between 1998 and 2014, there was a steady increase in the number of non-cervical genital cancers, specifically cancer of the anus, of the vulva and of the penis. This increase was more prominent in women than in men.

**Table 1:** Burden of HPV-associated cancers in Colombia

Annual number of cervical cancers cases		4661
Annual number of cervical cancer deaths		1986
Crude incidence rates (x/100,000 per year)	Male	Female
Cervical cancer	–	19.3
Anal cancer	0.2–1.3	0.8–1.9
Vulvar cancer	–	0.5–1.0
Vaginal cancer	–	0.0–0.7
Penile cancer	1.1–2.2	–
Oropharyngeal cancer	0.8	0.3

Source: HPVcentre.net, fact sheet Colombia 2017. The ranges (where provided) represent the lowest and highest recorded rates in different regions of the country

Colombia contributed 1203 specimens to the ICO International survey on HPV-associated cancers (Table 2). Based on HPV prevalence studies, disregarding potential cross-protection, vaccination with the 2-valent or 4-valent vaccine could prevent 68% of cervical cancers in Colombia, and between 48 and 81% of the other HPV-associated cancers, whereas the 9-valent vaccine could prevent 88% of cervical cancers, and between 85 and 100% of the other HPV-associated cancers. These results are similar to those published previously, based on a similar dataset [14]. In H&N cancers, the second most frequent type of cancer related to HPV infection among Colombians, there are large differences in HPV prevalence between sub-sites, with HPV prevalence in oropharynx (62.5%) being much higher than in other sites (oral cavity 8.6%; larynx 19%).

**Table 2:** Potentially preventable fraction of HPV-associated cancers in Colombia with currently available HPV vaccines

Anatomic Site	No.	HPV DNA+	HPV DNA+ Bivalent (2-valent) or quadrivalent (4-valent) Vaccines	HPVDNA + Nonavalent (9-valent) Vaccine
ANUS	39	94.9%	81.1%	100.0%
CERVIX	841	78.4%	67.5%	87.7%
HEAD & NECK	192	26.0%	80.0%	88.0%
PENIS	54	50.0%	70.4%	85.2%
VAGINA	29	72.4%	47.6%	85.7%
VULVA	48	60%	79%	86%

*Based on HPV+ cases

Source: Hernandez-Suarez G, Muñoz, N et al. unpublished results

Based on the reduction in cervical cancer incidence, from a high-risk country with incidence rates above 30 per 100,000, Colombia has now become an intermediate-risk country, with rates about 15 per 100,000, although still leaving room for improvement. A similar reduction on mortality rates has been observed in the last 30 years, especially in middle and high-income groups and populations with easy access to health services. Large geographical differences in the mortality rates are observed in Colombia, with the highest rates in remotes areas with low density of population and difficult access to health services.

On the other hand, 5 years-survival rates for cervical cancer have been increasing during the last two decades in Cali, Colombia, but are still lower (46%) than those reported from the US (63%) and other developed countries [15].

Despite the evident decrease in mortality, there is still ample evidence to aim for improvements in HPV vaccination and cervical cancer screening. First of all, in view of the clear inequality in cervical cancer mortality with higher rates in women from low socio-economic groups and difficult access to health services, vaccination is a more certain way to achieve equality. Secondly, vaccination has the potential to decrease cervical cancer incidence to levels below that which can be reached by screening alone under the best circumstances. Ideally both vaccination and screening should be integrated into a cervical cancer preventive program.

## Prevention and control of HPV and HPV-related cancers in Colombia

### The cervical cancer screening program in Colombia

**Fig. 1:**
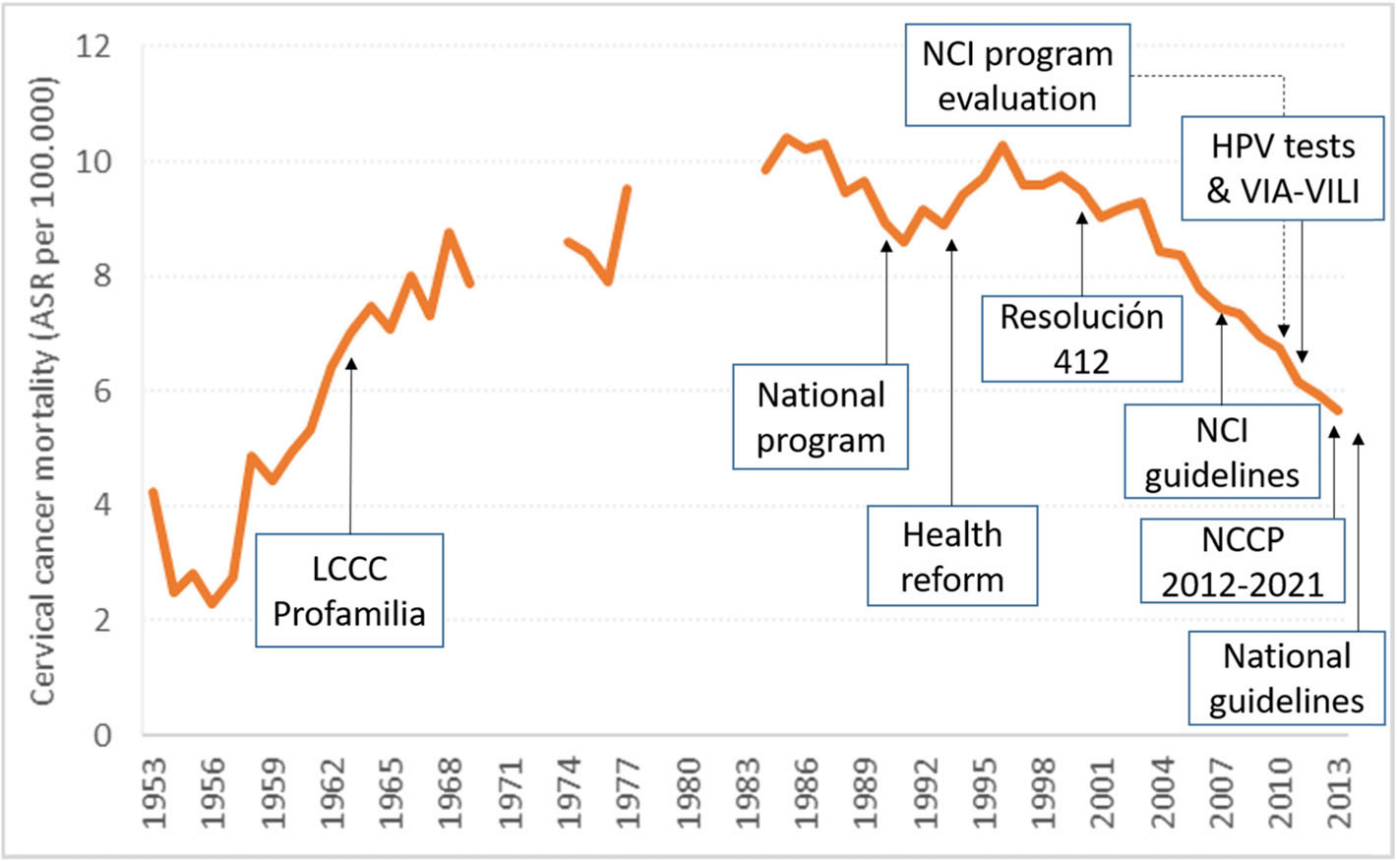
Cervical cancer screening timeline and cervical cancer mortality in Colombia

Cervical cancer screening was introduced in the 60’s and was implemented by two NGOs: the Colombian League against Cancer and Profamilia; however, due to its non-organized nature and low participation, this proved to be non-effective. In the 90’s screening was reintroduced as a National Program under the coordination of the National Cancer Institute of Colombia which promoted the practice of Pap smear outside the maternal program to reach high risk populations, and in 2000 it was regulated based on governmental Resolution 412, which led to a decrease in cervical cancer incidence [Fig. 1]. The reductions in cervical cancer incidence, however, have not been uniform across the population due to differential follow-up of an abnormal Papanicolaou (Pap) test based on women’s health insurance: greater follow-up of abnormal Papanicolaou test results was found for women with pay-roll insurance while sub-optimal follow-up of abnormal results occurred for women without insurance as well as those with subsidized insurance [16]. The difference in follow-up care following abnormal cervical screening results has resulted in differences in mortality due to cervical cancer: women with pay-roll insurance had lower mortality rates than women with subsidized or no health insurance. No differences were observed between women with subsidized insurance and those without insurance in any age group, indicating that subsidized insurance did not provide protection against cervical cancer mortality compared to no insurance [17, 18].

A new Resolution, number 3280, is in progress, introducing cytology from 25 to 30 years of age, HPV testing from 30 to 69 years, and screen-and-treat approaches for areas with low access to health care as defined by the Ministry of Health. The new resolution is expected to result in fewer screening visits for women and creates an established timeframe between colposcopy and treatment of less than 30 days. The new program will be monitored to see if there is an impact on the number of women with cervical precancer high-grade squamous intraepithelial lesions [HSIL], a cytological diagnosis, cervical intraepithelial neoplasia [CIN]2/3, a histological diagnosis, or adenocarcinoma in situ. Shortcomings of the program seem to be lack of clear stewardship with no collaborative plan between territories and insurers, lack of organized quality assurance, and unjustified risk of screening adolescents as allowed in the new regulation, for whom detection of transient HPV infections may lead to unnecessary treatment.

### The HPV vaccination program in Colombia

In Colombia, HPV vaccines were authorized before national policy recommendations; within the context of the decentralized decision-making process, HPV vaccination activities were driven by pressure from local political actors [19]. Parents’ acceptability of vaccination in Colombia varied according to socio-cultural context; in some regions preventing a sexually transmitted infection amongst parents of very young girls (< 12 years) hamper their acceptability [20].

Introduction of the HPV vaccine into national immunization program was prepared in close cooperation with Colombian scientific societies. In 2008, a cost-effectiveness study was performed [21], which led to the introduction of the HPV vaccine into the national immunization plan in August 2012, at the same time as the hepatitis A vaccine. The 4-valent vaccine was chosen, because of the added protection against genital warts due to the inclusion of HPV 6/11. The target population was girls in 4th grade of primary school and aged 9 years and older, to be vaccinated in a school-based system. Soon after vaccine introduction, in 2013 the vaccination schedule was changed from 3 doses at 0, 2 and 6 months to 0, 6, and 60 months in the hope that the dose at 60 months would not have to be given, as initial studies began to show the benefits of a two-dose schedule. The reduction in number of doses allowed a catch-up program, aimed at girls between 9 and 17 years of age, resulting in a 1st dose coverage of 94.8% and a 2nd dose coverage of 76.6%. This catch-up program was implemented only in 2013.

In 2014, and thereafter, the target population was again girls in 4th degree of primary school older than 9 years of age. In June 2014, reports of adverse symptoms (headaches, paresthesia, and back pain) were received from girls residing in Carmen de Bolivar who received the 2nd dose of the HPV vaccine. After a few days, similar symptoms were reported from more than 600 girls. This news was in the headlines for 2 weeks. Despite studies showing that there was no correlation with vaccination, a big drop in coverage ensued, to 60% in 2015 and to 6% in 2016. In addition, the National Academy of Medicine recommended that a screening for autoimmune diseases be performed before the administration of the HPV vaccine. Due to these events, school-based vaccine programs were discontinued.

To increase vaccine confidence, a media campaign was initiated, involving high-level medical figures, which resulted in a slight increase (17.6%) in vaccine coverage in 2018.

In April 2018, the Ministry of Health decided to change the HPV vaccination scheme to two doses (0 and 6 months), for all immunocompetent girls between 9 and 18 years of age. For optimal impact of the vaccination program, the following has been considered necessary: 1) acceptance of the vaccine by the adolescents/teenagers and their parents; 2) appropriate knowledge and understanding of the vaccine by healthcare workers; 3) correct management of information about vaccine safety; and 4) immediate response to rumors about the vaccine [22–23].

### Vaccine surveillance in Colombia

In 2008 the Colombian National Institute of Health and the Colombian Institute of Surveillance of Drugs and Food (INVIMA) started the Eventos Supuestamente Atribuibles a la Vacunación o Inmunización (ESAVI) program to perform the surveillance for potential vaccine adverse events in Colombia. This surveillance system is one of the strongest in America, it is nominal or individual and it is based on weekly reports from nearly 5200 health institutions, representing 1117 municipalities [22]. In addition, it also includes information from 6300 other sources. All events are reported in which a patient presents with any disorder, syndrome, sign or symptom that might be related to the administration of a vaccine, with a maximum delay of 4 days. This includes injection site abscesses; events that require hospitalization; events that cause disabilities; death occurring within 4 weeks after the administration of a vaccine, and potentially related to vaccination; events that are above the expected incidence in the population; and any rumor about the safety of a vaccine that is generated in the media. These events (or rumors) are investigated and classified as: 1) a case related to the vaccine - the event has been described as a reaction to the vaccine due to its components, fulfilling the causality criteria; 2) a programmatic error – a case in which it is possible to identify a failure in the application, storage and distribution of the vaccine; 3) a coincidental case – an event that occurred at the time of administration of the vaccine, without a clear relationship to the vaccination; 4) inconclusive event - an event in which not all the necessary information is available to establish if the event was caused by the vaccine.

For the HPV vaccine, the Carmen de Bolivar incident resulted in 240 adverse events (AEs) per 100,000 doses in 2014 and thereafter the reported rate of adverse events decreased gradually, to a level similar to other vaccines, 7–8 events per 100,000 doses. From 2012 to 2018, only one severe AE was reported: a case of complicated urticaria, in which case the subject was hospitalized. All other cases were considered as mild and/or not related to the HPV vaccine.

### Potential immune-mediated disorders

Surveillance is performed worldwide in all countries that have introduced HPV vaccination.

As vaccinations are common, even a small increased risk of multiple sclerosis (MS) or other acquired central nervous system demyelinating syndromes (CNS ADS) could have a significant effect on public health. However, no long-term association of the HPV vaccine with MS or any other CNS ADS was found, arguing against a causal association [23, 24]. Similarly, in a nationwide register-based open cohort study in Sweden, HPV vaccination was not associated with an increased incidence of new-onset autoimmune disease in girls and women with pre-existing autoimmune disease [25], which is relevant for the Colombian situation, as currently, testing for autoimmune diseases is advised before vaccination. Looking for incident hospital diagnosed autoimmune, neurological, and venous thromboembolic events (53 different outcomes) up to 180 days after each HPV vaccine dose, no evidence supporting associations between exposure to HPV vaccine and these events was found in Denmark and Sweden [24]. Although associations for three autoimmune events were initially observed, on further assessment these were weak and not temporally related to vaccine exposure. In contrast, inverse associations were found with epilepsy (rate ratio 0.66, 95% confidence interval 0.54 to 0.80) and paralysis (0.56, 0.35 to 0.90) [26].

Findings from a qualitative review of 14 safety studies on CNS ADS, MS, and optic neuritis strongly supported the absence of an association between HPV vaccines and central demyelination [27]. Owing to limited data on Guillain-Barré syndrome (GBS), no meta-analysis could be performed for this outcome. Data from French nationwide databases suggested that the incidence of GBS was increased among vaccinated girls (incidence rate of 1.4 among vaccinated [20 cases] versus 0.4 per 100,000 person years among unvaccinated [23 cases]; adjusted hazard ratio: 3.78 [1.79–7.98]). Under the hypothesis of a causal relationship, this would result in 1–2 GBS cases attributable to HPV vaccine per 100,000 girls vaccinated [28]. However, based on Hospital Episode Statistics (HES), no evidence of an increased risk of GBS was found following HPV vaccination in England. Based on the upper end of the 95% CI for the relative incidence and the number of HPV vaccine doses given in England, the data can exclude a risk of about 1 per million doses [29].

Similarly, for postural orthostatic tachycardia syndrome (POTS) the Vaccine Adverse Event Reporting System (VAERS) database for reports of POTS following HPV vaccination 2006 to 2015. Among 40,735 VAERS reports, 29 POTS reports that fully met diagnostic criteria were identified: approximately one POTS case per 6.5 million HPV vaccine doses. No unusual or unexpected reporting patterns was detected that would suggest a safety problem [30]. These combined data led the Global Advisory Committee on Vaccine Safety of the World Health Organization (GACVS) to conclude that since licensure of HPV vaccines, GACVS has found no new adverse events of concern based on many very large, high quality studies and therefore GACVS considers the HPV vaccines to be extremely safe [31]. Nevertheless, in Japan, HPV vaccines became part of the national immunization program in April 2013. In response to media coverage regarding possible adverse events, the Japanese government suspended proactive recommendation of its use in June 2013. In total, 2584 adverse events were reported, out of a total of 8.9 million HPV vaccine doses (0.03% of total doses) given to 3.38 million persons (0.08% of total persons) [32]. Of those reporting adverse events, approximately 90% complete recovered, 186 persons still received medical care, 3 died (due to suicide, neoplasm and cardiac disease) and 2 people had long-term health effects. At the same time, the mortality rate from cervical cancer in Japan increased by 3.4% from 1995 to 2005 and is expected to increase by 5.9% from 2005 to 2015, in particular among women aged 15–44 years [33].

In conclusion, the issues of safety as well as the need for the HPV vaccine must be addressed with healthcare workers, adolescents and parents. HPV vaccines were developed to prevent cancer and save lives. To this effect, it may help to train (future) healthcare workers (HCWs) to provide a strong recommendation of the vaccine.

### Monitoring effectiveness of HPV vaccination in Colombia

To evaluate the effectiveness of the HPV vaccination program, type-specific HPV prevalence was compared between 1451 vaccinated and 951 non-vaccinated women aged 18–25 years old, from a single Colombian city, 5 years after vaccination [34]. Significant reductions were observed in all vaccine types, with a reduction of 74% for HPV 6, 100% for HPV 11, 73% for HPV 16 and 55% for HPV 18. Furthermore, limited reductions in HPV types 31 and 45 were observed in vaccinated versus unvaccinated women (although not statistically significant), suggesting that the vaccine might provide some degree of cross-protection against non-vaccine high-risk HPV types. Results have shown that optimal benefit of vaccination was obtained when women were vaccinated with a complete vaccination schedule, before sexual debut.

## Successes and challenges in other countries/regions

### Brazil

Brazil is a large country with great geographical differences between regions. The target population for HPV vaccination comprises 17 million boys and girls, aged between 9 and 14 years. Cervical cancer is the 3rd most frequent cancer among women, with an incidence of 15.4 cases /100,000, and 5000 deaths per year.

From 2014, HPV vaccination was introduced in a scaled-up fashion, first targeting 11–13-year-old girls, in a 3-dose schedule, followed in 2015 by vaccination of males and females living with Acquired immunodeficiency syndrome (AIDS). In 2016, the target age for girls was expanded to 9–14-year-olds and beginning in 2017, boys were targeted between the ages of 11–14 years, as well as males and females, aged between 15 and 26 years, with weakened immune systems. Since the start of the vaccination program, 37.5 million doses of vaccine have been given.

To establish a baseline for evaluation of the effectiveness of HPV vaccination in Brazil, the POP-Brazil study [35] is investigating HPV prevalence and genotypes, in sexually-active men (*n* = 1774) and women (*n* = 5812), aged 16 to 25 years. 35% of participants had a high-risk infection and 31% had an infection with more than one HPV type. In September 2014, 80 girls presented with symptoms (paresis, numbness, temporary legs paralysis, similar to those seen in Colombia. All were hospitalized but recovered spontaneously. The authorities judged the symptoms to be related to immunization anxiety. Nevertheless, this situation had a negative impact on vaccine coverage. In total, between 2014 and 2018, 163 serious AEs were reported, with an average incidence of 0.67 cases per 100,000 doses.

Several steps were considered important to improve vaccination coverage in Brazil, including: improving the interface between health and education sectors, especially the health in school program; introducing/enforcing an immunization requirement for school entry; improving the quality of information on, and evaluation of, the vaccine; instituting partnership with scientific societies and civil society; progress in the operation of the National Immunization Program Information System throughout the country.

### Peru

As one of the countries in the Merck-PATH study, the HPV vaccine was introduced in Peru in 2008. This study showed that school-based vaccination leads to the highest vaccine coverage. Peruvian parents were very positive about vaccines, believing strongly in the value of vaccines. Furthermore, vaccines were provided for free and recommended by the Minister of Health. The main reason for not being vaccinated was absence of school at the day of vaccination; neither fear of early sexual debut nor adverse events were mentioned as reasons for not being vaccinated.

Although the Peruvian president announced that HPV vaccination would start in 2008, it was only in 2011 that a ministerial resolution was released. Defining age of vaccination at 10 years, 280,000 girls were going to be vaccinated with the bivalent vaccine. However, the vaccination program was not budgeted, and no vaccination database was available. Therefore, active immunization at schools, as part of the initial strategy, was stopped. In 2015, the 4-valent vaccine was bought with the help of UNICEF and the Pan American Health Organization. This led to an increase in first dose vaccine coverage to 77%, similar to what was achieved in 2011, whereas 3-dose coverage reached 66%.

Several measures were taken to increase coverage of 4-valent human papilloma virus (4vHPV) vaccine: the vaccination scheme was changed from three to two doses; a training program for health care workers about HPV vaccination was initiated; training was organized for directors of education and directors of schools in 24 regions on HPV and HPV vaccine; strategies were developed to involve parents and make it possible to obtain parental consent through the Internet; and a communication campaign was started, involving politicians and the media, and having a TV actress as the face of the campaign. Finally, a contest was held between 24 regions, to see which region was going to be the champion for a new generation of women without cervical cancer.

Future points of attention to further improve vaccination coverage are underway including development of an electronic vaccination card; facilitating electronic access for mothers to obtain their children’s vaccination records; changing vaccination informed consent from opt-in to opt-out; and consideration of catch-up, gender-neutral, and 1-dose vaccination strategies. Furthermore, measurement of vaccine impact is being considered.

### Panama

In 2008, Panama began a school-based HPV vaccine program, administering 2-valent HPV (2vHPV) vaccine, given at the age of 10 years according to a 3-dose schedule (0, 1, and 6 months). Beginning in 2015, the 4-valent HPV vaccine was used instead of 2-valent HPV. If the mother took her daughter for vaccination, she would be offered a Pap test herself. That same year, catch-up vaccination was offered to girls aged 16–17 years, in higher-risk regions, using the remaining 2vHPV doses. In 2016, the 4vHPV was also offered to boys from 10 years of age. In 2017, the vaccine coverage was 69%, which was relatively low, due to lack of 4-valent HPV vaccine stock, not due to AEs and its consequences. The vaccination crisis in Colombia was covered in the news, but there was a quick response in Panama to any anti-vaccine movement, with journalists involved. The Panama experience demonstrated that a political decision is needed to effectively set up HPV vaccination.

### Ireland

In Ireland, a school-based HPV vaccination began in 2010 targeting 12–13-year-old girls (approximately 30,000 per year). In the first 5 years, vaccine coverage was very high, but in 2015 there was a decline in coverage, followed by a massive drop in 2016. Some parents raised major concerns about the vaccine. They were active on social media as well as TV. In response to this, the Health Service Executive (HSE) Ireland developed a website: www.hpv.ie [36], with information on HPV and the vaccine, and had the website accredited by the WHO.

Furthermore, the HPV Alliance was established in August 2017, which now has 37 member institutions, including healthcare workers as well as women’s and children’s groups, all promoting the vaccine. Fact sheets were prepared, for schools, general practitioners and parents. HSE took up social media listening, to be aware of the rumors going on. In response to the rumors, HSE became active on social media, including twitter, using the hashtag #protectourfuture. In all of this, the Health minister was very supportive. And as a positive outcome, vaccine coverage increased from 50 to 65%, which is still lower than before the crisis, but going in the right direction.

## Achievements and challenges in Colombia

### Nariño region

This region, along the border of Ecuador, has the highest vaccine coverage in Colombia. Where it aimed for a 17% increase, it managed to accomplish an increase to 47%.

This success was due to a number of actions: audits of healthcare workers, including education on HPV vaccination, both in public and private IPS; actively going out to rural areas with low access to healthcare to provide vaccinations; organizing peer-to-peer discussions on HPV vaccination in schools; using conventional and non-conventional media, including local radio stations to increase awareness; as well as, allocating a specific nurse for follow-up of women with abnormal Pap smear results, to guarantee treatment when a lesion was found.

### Carmen de bolivar

In 2014, before the crisis, vaccine coverage was 100% in 4198 girls. However, after the 2nd dose, the first cases appeared. A few days later, more than 600 girls started to have the same symptoms (headaches, paresthesia, back pain). Many hypotheses have been formulated to explain these symptoms, but once vaccination was raised as option, there was little attention for other potential explanations. In fact, the advocate leading the anti-vaccine movement approached the doctor in charge of two girls who were being attended at the hospital, with the request to change the diagnosis to syncope induced by the HPV vaccine.

An independent study carried out by the National Institute of Colombia ruled out a direct relation to the vaccine, but this led to much abuse towards spokespeople of the vaccination program, as it was interpreted as a callous denial of the symptoms of the girls.

Currently, 4 years later, everything has calmed down. Sixty-five girls of the group with symptoms are still being followed closely, but they have not had further crises. In order to be able to provide the best possible care for these girls, a mobile unit is being used to reach those girls in remote areas, who have limited access to healthcare. Furthermore, meetings with the parents have been organized.

### Manizales

In this region, 98% of the population lives in urban areas, and the population has a high level of education. To identify barriers and facilitators for HPV vaccination, a population-based survey was performed on girls born in the years 2003–2005, as well as their parents. Vaccination status was defined by girls’ report, parental report, and the online official vaccine registry. This showed good concordance between girls’ self-report of vaccination and official data. Their school was an important source of information for those girls who were aware of the vaccine. Importantly, 40–60% (unvaccinated) to 70–90% (vaccinated) of the girls were aware that the vaccine prevents cervical cancer and HPV infection, respectively, and these were strong reason for uptake of the vaccine. Nevertheless, 40% of the girls that were vaccinated thought the vaccine may be dangerous and produces AEs.

The most important barriers for vaccination were recommendations against vaccination by relatives or friends, as well as media attention related to AEs in Carmen de Bolivar or in other countries. Finally, not being offered vaccination in the school was an important barrier to completion of the vaccine schedule: these girls started but often did not complete the vaccine series.

## SWOT analysis of HPV vaccination in Colombia

### Strengths

Extensive knowledge of the epidemiology of HPV is available in Colombia, due to participation in scientific studies. Due to these studies, there is appreciation of the burden of cervical cancer in Colombia and a sense that country’s scientists played a key role in the development of the HPV vaccine.

Colombia has almost universal insurance coverage and an extremely effective immunization program for all vaccines. A strong surveillance program is available, which is necessary to monitor safety of the vaccination program.

There is strong political support and political will to continue and expand the HPV vaccination program, with consensus among different stakeholders on safety and effectiveness of the vaccine. The HPV vaccination program is a well-structured program, which, despite low vaccine coverage, is equitable. The vaccines are affordable at the country-level, and free for those targeted by the program.

### Weaknesses

HPV vaccination in Colombia is hindered by fragmentation in communication and continues to recover from a slow reaction to the Carmen de Bolivar crisis. In general, a communication plan for dealing with crises is lacking and there is inadequate capacity for communication with different groups (HCWs, teachers, general population), leading to a lack of HPV / cervical cancer education of HCWs, the public and media. Moreover, communication, especially with the public, is at the wrong level, being too complex and too scientific.

Following the Carmen de Bolivar crisis, Colombia abandoned the school-based system for HPV vaccination, while it has been shown to be the most effective system to achieve high coverage. Finally, HPV vaccination and cervical cancer screening have not been appropriately integrated.

### Opportunities

The recent reports on safety of the vaccine provide an opportunity to bring the vaccine under the attention of the media and the public in a positive way, aiming to increase vaccine coverage. Furthermore, the adapted E-learning course on vaccine safety (see below) is an excellent opportunity to educate HCWs on HPV and cervical cancer. Given the extensive experience from neighboring countries with the HPV vaccination program, Colombia can learn from these countries, copying successful strategies and avoiding pitfalls.

Finally, it would be appropriate to try to show sympathy to those affected by alleged AEs, regardless of whether these reactions are directly linked to vaccination. This is likely to increase the respect and appreciation of the public for the vaccination program and the HCWs involved.

### Threats

The geographical distribution of the Colombian population is a potential threat to the vaccination program: a part of the population has very limited access to healthcare. Furthermore, in some parts of the country, the social situation is (still) unstable. And there is an increasing burden on the healthcare system due to immigration.

Moreover, frivolous, predatory litigation towards the HPV vaccination program is likely to harm vaccine coverage, a problem that is aggravated by the strong activism of anti-vaccine groups in Colombia. This anti-vaccine sentiment may spillover to other countries and to other vaccine programs in Colombia and may lead to a higher burden of cervical cancer and other diseases due to reduced vaccine coverage.

Finally, there is uncertainty about political support in higher political circles, some opinion leaders are against the HPV vaccine and the sustainability of the HPV vaccination program is not guaranteed.

## SWOT analysis of cervical cancer screening in Colombia

### Strengths

Extensive knowledge, and appreciation, of cervical cancer is available in Colombia, due to participation in scientific studies. Furthermore, there is strong political will to eliminate cervical cancer as well as technical leadership, which can be shared with others in the region.

### Weaknesses

Colombia does not have a population-based program, leading to disparities and difficulties in access to screening. Furthermore, there is a lack of surveillance and no program for follow-up of abnormal Pap smears, which prevents screening from providing any added value. Finally, changes in the screening program cannot be introduced due to regulatory stifling.

### Opportunities

The new technologies for HPV detection may provide an opportunity to boost cervical cancer screening in Colombia. This is also an opportunity to integrate vaccination and screening. Furthermore, using different approaches for different populations (e.g. VIA [visual inspection after acetic acid], self-sampling), screening may become more equitable.

New legislation is coming up, integrating different actions. Finally, an excellent tool has been developed to train the healthcare workforce. E-oncologia (http://www.e-oncologia.org) [37] is a virtual oncology training program, developed by the Catalan Institute of Oncology (ICO), with 71 courses and 2300 h of training materials, in 9 languages. One part of the programme is devoted to cervical cancer prevention. As trainees can become tutors, a snowball effect in training is envisioned.

The module can be calibrated to local needs, such as an adapted course for Colombia after the vaccine crisis, with a focus on the safety of the HPV vaccine. This course, jointly developed by the ICO and the Colombian Cancer Institute, is directed towards doctors, nurses and/or midwives, and in general all health professionals involved in the primary and secondary prevention of cervical cancer. The chapters have been co-authored by Spanish and Colombian experts. So far, close to 5000 participants (of whom > 80% are Colombians) have registered, of whom OVER 70% have completed the course and completed the final exam successfully.

In fact, the course could possibly be adapted to be used for non-medical people, such as journalists.

### Threats

The geographical distribution of the Colombian population is a potential threat to the screening program, a part of the population has very limited access to healthcare. Next, in some parts of the country, the social situation is (still) unstable and, owing to the political crisis in Venezuela, there is an increasing burden on healthcare system due to immigration. The cost of screening is very high and failure to reach an agreement to implement new techniques may further hamper the screening program. Finally, there is no systematic roll-out of information to the public.

## Colombian stakeholders

In response to the Carmen de Bolivar crisis, the ‘Mesa de Concertacion’, a roundtable for the elimination of HPV-related cancer in Colombia, was organized. This roundtable brought together many stakeholders: the Colombian League against Cancer; Profamilia; universities; scientific societies (including pediatrics, gynecology, family medicine, oncology); the association of cities; the National Academy of Medicine; parent associations; the Ministry of Health; pharmaceutical and diagnostics industry; as well as foreign organizations, including the American Cancer Society. Actions following from the roundtable include education to healthcare providers, regaining confidence in the vaccine by giving lectures throughout the country, and reactivation of the school-based system.

## The role of media and journalists

Many believe that there is a trend towards sensation journalism, exacerbated by social media, where everyone can be a journalist. A bit of background helps to understand the media better: national and regional media often have very different editorial policies of accepting topics for dissemination. Furthermore, the way news is covered depends on more than just the journalist: the inclusion of photos; the impact of the layout; the editor, the choice of title, all aspects that may create a completely different perception of the news.

Scientists often distrust journalists, but it is necessary to acknowledge that journalists are actually very powerful and have a large outreach. Therefore, scientists need to reach out the journalists, with factual information, keeping in mind that if journalists receive accessible and clear information, they are more likely to transmit them to the public. A relationship with the media is not a one-time affair, it is a permanent relationship, that needs to be nurtured. Public health actors must consider journalists their communication partners.

It is the duty of a journalist to discuss both sides of a story, but sometimes this results in an incorrect balance; it may help to point this out, showing the potential negative consequences (e.g. deaths due to non-vaccination) of publishing a news item. Well-trained media personnel will make fewer mistakes. While there are master’s programs in scientific journalism, science is a very broad topic, much broader than just health, so specific education for journalists remains necessary.

Finally, media can be an important factor in discrediting the vaccine. If a good relationship with a journalist is established, he/she could point out how other journalists can be reached, to make them allies, avoiding unnecessary damage to vaccination programs.

## Way forward

After one and a half days of interesting presentations and lively discussion, it was concluded that there is still a lot of work to do. Therefore, several action points have been formulated that can be pursued to increase vaccination coverage in Colombia:

- Re-introduce the school-based system for HPV vaccination, as it has been shown to be the most effective system to achieve high coverage.
- Adoption of Resolution 3280 might be a good time to integrate primary and secondary prevention programs, as both have the same overall goal: reduction of cervical cancer. This should be done with a focus on equity and sustainability of the programs.
- Develop a communication plan for crisis situations and adequate capacity for communication with different groups (i.e. HCWs, educators, whole population). While the e-course is a good tool for training of HCWs, different ways of educating teachers and the general population need to be developed To this respect an alliance is being organized with the local office of the Open University of Catalonia (UOC), a university with a long tradition in e-learning educational programs in Spanish language to support the initiatives of the national cancer institute
- Establish a good and long-lasting relationship with the media, trying to make them a communications partner for the vaccination program.
- Bad news is swiftly communicated widely; it is time to start spreading the good news, at an accessible, non-technical level, for everyone to understand. Good news encompasses safety of the vaccine, as shown in a plethora of studies worldwide, as well as impact of the vaccine on HPV infections and HPV-associated lesions, shown in countries with high vaccination rates (e.g. Australia), but also in countries with lower vaccination rates such as the United States.
- Show empathy, public health officials must show respect and acknowledge the symptoms of those affected by them, also if these are not vaccine related. The plea from families of alleged victims must not be ignored. Medical Doctors (MDs) and nurses who sympathize will eventually find an opportunity to communicate with victims and their families and better understand their problems and concerns.

## Post meeting Progress report

The overall country HPV vaccine coverage rates increased slightly the year following the meeting from 29 to 34% for the first dose and from 9 to 11% for the 2nd dose. This increase was the result of an increase in a few regions such as the departments of Antioquia and in Arauca, where a special project is being implemented. In other departments, (Nariño and Valle del Cauca), the rates remain stable or have decreased. (Table 3). To increase coverage of the 2nd dose a delayed second dose strategy is being discussed; currently it is being administered 6 months after the 1st dose.

**Table 3:** HPV vaccine coverage rates in Colombia

Selected Departments	1st dose - 2018	1st dose - 2019	2nd dose - 2018	2nd dose - 2019
Arauca	41%	83%	11%	24%
Antioquia	39%	48%	12%	19%
Nariño	57%	53%	33%	32%
Valle del Cauca	48%	54%	35%	21%
Total Colombia	29%	34%	9%	11%

Source: Information provided by the PAI, MSPS- Ministry of health of Colombia

### The Arauca project

After the collapse of the National HPV vaccination program following the Carmen de Bolivar event, the Colombian League against Cancer, initiated a project in Arauca in June 2018 to regain confidence on the HPV vaccine, in collaboration with the Colombian Ministry of Health, the American Cancer Society and Johns Hopkins University. Arauca is the Colombian department with one of the highest mortality rates from cervical cancer (11.4 per 100,000) and one of the lowest HPV vaccination rates. A new communication strategy was developed from a bio-psycho-social perspective to educate and sensitize girls to be vaccinated, the parents of the girls, the teachers, health care providers and the local mass media on the benefits and safety of the HPV vaccine. Eighteen months after starting the project, the coverage rate for the 1st vaccine dose in Arauca increased from 4.7% in 2017 to 83% in December 2019. Most of the increase in the 1st dose occurred in the last 6 months of 2019, thus coverage of the 2nd dose will be best measured in the first 6 months of 2020. We hope to reproduce this strategy in other regions of the country.

## Abbreviations

HPV: Human Papillomavirus
EPS: Entidades Promotoras de Salud
IPS: Instituciones Prestadores de Salud
IARC: International Agency for Research on Cancer
ICO: Catalan Institute of Oncology
H&N: Head & Neck
NGOs: Non – Governmental Organizations
Pap: Papanicolaou
HSIL: High-grade squamous intraepithelial lesions
CIN: Cervical intraepithelial neoplasia
INVIMA: Colombian Institute of Surveillance of Drugs and Food
ESAVI: Eventos Supuestamente Atribuibles a la Vacunación o Inmunización
AEs: Adverse Events
MS: Multiple sclerosis
CNS ADS: Central nervous system demyelinating syndromes ()
GBS: Guillain-Barré syndrome
HES: Hospital Episode Statistics
POTS: Postural orthostatic tachycardia syndrome
VAERS: Vaccine Adverse Event Reporting System
GACVS: Global Advisory Committee on Vaccine Safety of the World Health Organization
AIDS: Acquired immunodeficiency syndrome
4vHPV: 4- valent human papillomavirus
2vHPV: 2- valent human papillomavirus
HSE: Health Service Executive
HCWs: Healthcare workers
VIA: Visual inspection after acetic acid
UOC: Open University of Catalonia
MDs: Medical Doctors
